# Experts' perception of support for people with dementia and their families during the COVID‐19 pandemic

**DOI:** 10.1111/ggi.14307

**Published:** 2021-11-09

**Authors:** Kana Kazawa, Masahiro Akishita, Manabu Ikeda, Takeshi Iwatsubo, Shinya Ishii

**Affiliations:** ^1^ Department of Medicine for Integrated Approach to Social Inclusion Graduate School of Biomedical and Health Sciences, Hiroshima University Hiroshima Japan; ^2^ Department of Geriatric Medicine Graduate School of Medicine, The University of Tokyo Tokyo Japan; ^3^ Department of Psychiatry Osaka University Graduate School of Medicine Suita Japan; ^4^ Department of Neuropathology Graduate School of Medicine, The University of Tokyo Tokyo Japan

**Keywords:** COVID‐19, dementia, infection control, prevention

## Abstract

**Aim:**

This study aimed to explore the perceptions of dementia experts on support for people with dementia (PWD) and their families, considering PWD's vulnerability regarding COVID‐19 prevention.

**Methods:**

A collaborative qualitative study was conducted, involving Hiroshima University, the Japan Geriatrics Society, the Japan Society for Dementia Research, and the Japanese Psychogeriatric Society. An anonymous, self‐reported questionnaire survey was sent to dementia experts from 456 medical centers for dementia in Japan. The responses were categorized in a qualitative inductive manner.

**Results:**

A total of 214 experts from 119 centers responded (facility recovery rate: 26.1%). Four core themes emerged from the data analysis. Of these themes, three were related to support for infection prevention and related issues and response to infection: (i) support for continuation of daily life while preventing infection; (ii) support to mitigate the unfavorable effects of infection prevention measures; and (iii) decision‐making support and treatment for infected PWD. The remaining theme, (iv) community building for PWD living together, was extracted as a basis for facilitating themes (i) to (iii). Furthermore, in each theme, the roles of medical and long‐term care facilities, administration, and the need for community collaboration were identified.

**Conclusions:**

Dementia experts strongly felt the need not only for short‐term support to prevent the spread of infection to PWD and their families during the pandemic, but also for long‐term support to enable them to maintain their daily lives and mitigate the impact of infection prevention measures. **Geriatr Gerontol Int 2022; 22: 26–31**.

## Introduction

In late 2019, the novel coronavirus (SARS‐CoV‐2) emerged in Wuhan, China, and was identified as the cause of coronavirus disease 2019 (COVID‐19).^1^ Since then, COVID‐19 has spread across the globe, and the first Japanese patient was diagnosed in January, 2020.^2^ Older individuals and those with comorbidities have been reported to have a high mortality risk from COVID‐19.^3^, ^4^ Furthermore, current data suggest that dementia is considered to be an independent risk factor for COVID‐19 mortality.^5^, ^6^

COVID‐19 spreads among humans mainly by contact with droplets of mucus and saliva, and physical distancing and the wearing of face masks are recommended as infection control measures to avoid transmission of the virus.^7^ However, PWD experience considerable difficulties in implementing infection control measures. First, some PWD may be unable to understand the risks of infection and may fail to follow recommendations for infection control owing to cognitive decline.^8^, ^9^ Furthermore, behavioral and psychological symptoms of dementia (BPSD), such as agitation and wandering, may undermine efforts to maintain physical distance from others. Second, those with more severe dementia may be unable to communicate the presence of any symptoms when they become infected.^10^ Combined with the tendency of older adults infected with COVID‐19 to present with atypical symptoms such as delirium and falls,^11^ this may cause delays in the diagnosis and treatment of infection. Furthermore, delirium is highly prevalent in patients with COVID‐19,^12^ making treatment for COVID‐19 even more difficult.^12^ Third, PWD may need support from informal caregivers such as family members and long‐term care insurance services to meet their daily needs and maintain their activities of daily living, including exercise and social interactions. Infection control measures, including physical distancing and self‐isolation by remaining at home, can disrupt their daily routines, reduce their interaction with the community, and lead to a decline in physical and cognitive functions, as well as to the onset or worsening of BPSD.^13^ Not only does this make it more difficult for PWD to comply with recommendations for infection control, it also leads to an increase in their families' anxiety and their care burden.^14^, ^15^

In Japan, which is a super‐aging society, the estimated prevalence of dementia among people aged 65 and over was 15.0% in 2012, and this is expected to increase to 20.8% and 21.8% by 2030 and 2050, respectively.^16^ Against the backdrop of the increasing number of elderly individuals with cognitive impairment, the Japanese government introduced a new national health program called the Medical Center for Dementia (MCD) in 2008. The MCD aimed to provide specialized medical care for PWD and to contribute to establishing a comprehensive support network for PWD, in collaboration with community resources (Table S1).^17^

Regrettably, with the possibility of a prolonged COVID‐19 pandemic, developing practical support for PWD and their families in terms of infection prevention and dementia care has become imperative. Therefore, this study aimed to investigate the perceptions of dementia experts affiliated with the MCD regarding support for PWD and their families, as the role of the MCD is to promote dementia care in the community and to play an important role in policymaking.

## Methods

### Participants and recruitment

Participants were dementia specialists or physicians who had been engaged in dementia care for more than 5 years and who belonged to the MCD. The questionnaires were posted to all 456 MCD facilities. The survey period was from July to August, 2020.

### Data and analysis

This qualitative study was conducted using a self‐administered questionnaire (Supplementary Material S1). The questionnaire consisted of basic attributes, and the questions developed to obtain comprehensive perceptions of dementia specialists at MCD facilities on the support for PWD and their families.

Therefore, the entire response was analyzed as a single data set, rather than the responses to each question being analyzed individually, and we used the following methods to ensure the credibility and confirmability of the study. First, all data were processed using KH Coder® text‐mining free software.^18^ The software classifies groups of words that occur together often in the same document into clusters using a multivariate analysis. This enables researchers to understand the classification and association of words without relying on their preconceptions or theoretical background. Second, the interview raw data were thematically analyzed, including generative coding and theorizing.^19^ The researcher extracted codes, bundled the codes from the context, and extracted themes. To ensure the quality of the results, the results were discussed and interpreted with researchers, including dementia specialists. This study adhered to the Standards for Reporting Qualitative Research (SRQR) guidelines.^20^

The present study was conducted by Hiroshima University in collaboration with the COVID‐19 response team of the Japan Geriatrics Society, the Japan Society for Dementia Research, and the Japanese Psychogeriatric Society. Approval by an ethics committee was not necessary, as the method of the study was an anonymous questionnaire survey. The questionnaires were posted to participants together with a document explaining the purpose of the study and assuring participants of the protection of their privacy and their freedom of participation. Participants returned their answers anonymously and were deemed to have provided consent to participate in the study upon submission of the questionnaire form.

## Results

The questionnaires were distributed to 456 facilities, and 214 dementia experts from 119 of those facilities responded (facility recovery rate: 26.1%). All data were analyzed; Table 1 presents the characteristics of the 214 participants. The most common type of MCD to which participants belonged was the regional type, and the most common types of medical facilities were psychiatric hospitals (45.8%) and general hospitals (43.5%), while clinics accounted for 7.0%. Regarding the participants' specialties, for the majority it was psychiatry (66.4%), followed by neurology (28.0%), and general internal medicine (3.3%).

**Table 1:** Characteristics of the study participants (*N* =  214)

Attributes		*n*	(%)
Type of medical center for dementia to which participants belonged	Core type	33	(15.4%)
	Regional type	149	(69.6%)
	Collaborative type	27	(12.6%)
	No response	5	(2.3%)
Type of medical facility to which participants belonged	General hospital	93	(43.5%)
	Psychiatric hospital	98	(45.8%)
	Clinic	15	(7.0%)
	Other (e.g., University hospital)	8	(3.7%)
Participants' clinical department (overlapping distribution)	Psychiatry	142	(66.4%)
	Geriatric medicine	5	(2.3%)
	Neurology	60	(28.0%)
	General internal medicine	7	(3.3%)
	Neurosurgery	2	(0.9%)
	Other (e.g., pulmonology, pediatrics, and geriatric psychiatry)	3	(1.4%)
	No response	3	(1.4%)

Our analysis identified four themes regarding infection prevention and related issues, response to infection, and community building, based on the participants’ descriptions. Below, we provide results for each theme. Furthermore, the roles of the following three positions were extracted for each theme: medical and long‐term care facilities; administration (government, prefectures, and municipalities); and communities (Table 2). A verbatim quote of answers by participants is indicated by quotation marks.

**Table 2:** Role of medical and long‐term care facilities, administration (government, prefectures, and municipalities), and communities in supporting people with dementia and their families

Themes	Role of medical and long‐term care facilities, administration (government, prefectures, and municipalities), and communities
Support for continuation of daily life while preventing infection	Medical and long‐term care facilities Provision of accurate and simple information on infection prevention based on their cognitive function and their level of daily living Flexible continuation of long‐term care services with infection prevention Establishment of a consultation service for PWD and their families Government, prefectures, and municipalities Educating about prevention of infection Support for families' care and their daily living Development of infection control guidelines and support for the continuation of local care services Improvement of inspection system
Support for mitigating the unfavorable effects of infection prevention	Medical and long‐term care facilities Guidance for PWD and their families to prevent deterioration of physical and cognitive function while staying at home, and routine functional and symptom assessment for PWD Medical care facilities Formulation of guidelines for medical and long‐term care staff to support the prevention of functional decline in PWD Linkage and consolidation of information with related organizations and communities (especially for high‐risk PWD) Psychological support for providers facing the risk of infection as front‐line workers and support for the continuation of outreach services Communities Continuation of informal services with physical‐distancing efforts Information sharing with residents and related organizations
Decision‐making support and treatment for infected PWD	Medical and long‐term care facilities Explaining to patients and their families the risk of functional decline associated with infection and isolation, and supporting decision‐making Prevention of the spread of infection and monitoring the infected Government, prefectures, and municipalities Linking information, mainly at public health centers, on infected people and medical care delivery systems
Community‐building for PWD living together	Medical and long‐term care facilities/Government, prefectures, and municipalities Educating about dementia and support for PWD, targeting the entire community as well as older adults specifically Communities Building familiar relationships within the community and continuing to watch over PWD by the community

PWD, people with dementia.

### Theme 1: Support for continuation of daily life while preventing infection

Dementia experts commonly acknowledged the importance of and difficulties in supporting PWD and their families to enable them to continue with their daily lives while preventing COVID‐19 infection.

#### Role of medical and long‐term care facilities

Participants recognized that it was important to communicate simply to PWD the risk of infection and recommendations on infection control measures using familiar tools such as the telephone, television, radio, public relations magazines and the internet, in a way that was suitable for their cognitive function: “First of all, we need to explain to them in a concrete and easy‐to‐understand way without using the words ‘refrain from going out’. For example, ‘taking a walk in the community is fine’, but ‘talking at close range for a long time is not’.” In addition, in order to provide long‐term care services without disruption, it was considered necessary to shift the current system to a more flexible one in which services could be provided face‐to‐face to a small group or individually, or remotely via the internet or telephone.

#### Role of administration

Participants expressed the need to raise awareness of the importance of correct infection prevention and to support families in maintaining their daily lives (e.g., providing direct support for daily care and financial support for families who take time off work to care for PWD): “Introducing care robots or setting up counseling services via the internet to reduce the care burden will be useful.”

In addition, the development of infection control guidelines and provision of supplies such as face masks and disinfectants to support the continuation of local healthcare services are essential. At the same time, maintaining the supply of services and improving financial support for healthcare providers and facilities – which would otherwise be forced to close or reduce services owing to widespread infection – was mentioned as an urgent issue. There is also a need for rapid and timely PCR tests for COVID‐19.

### Theme 2: Support for mitigating the unfavorable effects of infection prevention

Participants recognized that the adverse effects of long‐term infection control measures, such as a decline in cognitive and physical functions and the onset or worsening of BPSD, should be mitigated.

#### Role of medical and long‐term care facilities

Participants noted that it was important to periodically assess the cognitive and physical functions of PWD and to adjust their care in a timely manner to preserve their functions and quality of life.

In terms of medical care facilities, participants reported a need to develop guidelines for healthcare providers to support PWD and their families, and to coordinate information with relevant facilities to identify high‐risk patients at an early stage: close cooperation between MCD and relevant facilities is needed to identify those who need medical attention and early intervention. Further, psychological support for healthcare providers facing the risk of infection as front‐line workers and support for continuing outreach services were also emphasized.

#### Role of communities

The continuation of informal services such as dementia cafés (a place for PWD and their families to interact with local people and dementia experts) and group meetings in a way that conforms to physical distancing and other infection control measures was deemed to be essential for maintaining social interaction and peer support.

The need to share information about high‐risk individuals with dementia, such as those who are confined to their homes, those who live alone, or couples who both suffer from dementia, at the request of the relevant facilities and administration was addressed: “Establishing a system of cooperation between the community and relevant organizations to ensure that people in need of care can promptly access those services” was seen as important.

### Theme 3: Decision‐making support and treatment for infected PWD

Participants raised the concern that when a person with dementia is infected, decision‐making support for treatment is a difficult issue that requires ethical consideration.

#### Role of medical and long‐term care facilities

Healthcare providers are required to explain to PWD and their families the morbidity and mortality associated with COVID‐19 infection, including the possible decline in cognitive and physical functions and the methods and duration of isolation. They should also provide support for decision‐making related to these issues.

Consulting services on COVID‐19 infection control by infectious disease specialists and dementia specialists are needed: “As a regional care system, facilities should consult with specialists to consider in which hospital and which ward to treat patients according to their level of infection, severity of dementia, and BPSD.”

#### Role of administration

In Japan, public health centers have taken the lead in managing beds dedicated to the treatment of COVID‐19 and in tracing the route of infection. Therefore, a smooth and continuous information linkage between public health centers and medical and long‐term care facilities is required.

### Theme 4: Community building for PWD living together

To enable PWD to live their own lives in the community, local residents are expected to understand them and build relationships with them.

#### Role of medical and long‐term care facilities and administration

PWD who are unable to take appropriate precautions against infection may experience conflict within the community. In order to expand the understanding of dementia, raising awareness of dementia in the general community and among older people by using internet of things, public broadcasting, and lectures was recognized as important: “Support for people with mild dementia who have a lot of interpersonal contact is an issue, and it is necessary to gain the understanding of the local residents.”

#### Role of communities

To enable PWD to continue a secure life in their own way, they must be allowed to have social interaction in a familiar community; this was considered to be the basis for building relationships: “Individuals with dementia should build familiar relationships at a level close to their daily life, such as with people's welfare committees, neighborhood associations, and supermarkets.”

## Discussion

This study explored the perceptions of dementia experts who belonged to the MCD in order to support efforts related to the prevention of COVID‐19 infection and mitigate the associated unfavorable effects on PWD. As a result, support for infection prevention and community building for living with PWD emerged as a theme of high priority.

First, concerning “support for continuation of daily life while preventing infection,” the importance of explaining the risk of infection in terms of their dementia symptoms to PWD and their families and encouraging them to take individualized infection prevention measures and continue long‐term care was repeatedly mentioned. PWD have different symptoms, routines, and environments, and their risk of infection and methods of infection prevention are not uniform.^21^, ^22^ Considering individualized care and ensuring that daily life is as unobstructed as possible will reduce the burden on both PWD and families and provide a basis for the long‐term sustainability of prevention efforts.

Furthermore, regarding support for PWD and their families to maintain their daily lives, economic issues such as the costs of services and goods and the adjustment of working hours for long‐term care are also factors that affect the care burden.^23^ Therefore, it is necessary to consider public measures, such as financial support, when families need to take time off work for care or to purchase essential items such as masks and sanitizer, and in‐person care services.

Second, “support for mitigating the unfavorable effects of infection prevention” was extracted. This theme showed that dementia experts perceive that long‐term social‐distancing efforts could lead to a decline in cognitive and physical function and quality of life among PWD, as in previous studies.^24^, ^25^ Creating and supporting the use of various services, such as programs that stimulate cognitive functions and encourage physical activities and interactive online meetings, will undoubtedly reduce the unfavorable effects on PWD and enable them to continue with their own lives.

The third theme extracted, “decision‐making support and treatment for infected PWD,” was extracted as an ethical issue. This theme was considered a key factor in realizing the high quality of treatment and care desired by both the patient and his/her family. However, when a person with dementia becomes infected, changes in the environment due to hospitalization, stress from treatment, and hypoxemia, a prominent symptom of COVID‐19, can induce delirium. Delirium makes decision‐making difficult and worsens the prognosis.^5^, ^26^ In such critical conditions, families will also experience upset and conflict. Therefore, it is desirable for healthcare providers to create opportunities to discuss the expected trajectory of dementia and possible treatment options with PWD and families, not only in the case of infection, but also in preparation for the event of infection.^27^, ^28^ Furthermore, it is expected that when families are infected with COVID‐19, services to care for PWD on their behalf will become more widespread.

Finally, we extracted “community building for PWD living together” as a basis to promote the various supports summarized under the first three themes. Regardless of COVID‐19, this theme is considered fundamental to the lives of PWD and their families, with the community's understanding of dementia and support for PWD. During the pandemic, PWD and their families may be exposed to double prejudice, in addition to dementia‐related prejudice.^29^ This may affect the comfort of living in the community and accessibility to health and social services.^30^ Therefore, there is an urgent need for the entire community to disseminate knowledge about dementia and communication with PWD, and knowledge and skills on how to support PWD. Previous studies have shown that the more prejudiced the families themselves are toward dementia, the more they feel the care burden.^26^ In Japan, there is also a strong perception that it is natural for family members to care for PWD, and there are cases where the families are confined to their homes with PWD. It is therefore important to continuously develop effective support programs for families, including individual consultations by medical and nursing providers and peer support, from the early stages of the disease.

The findings of this study indicate that during the response to the COVID‐19 pandemic, dementia experts strongly expressed the need for both short‐term measures to prevent the spread of infection and long‐term measures to change the services and service delivery systems that support PWD and their families. Moreover, they emphasized the need to build an inclusive society where PWD can live their own lives, together with their communities. In the future, further investigation of how PWD and their families have experienced the COVID‐19 pandemic and an expansion of our understanding of dementia care will be required. There is also a need to further promote support for PWD and their families and to build an inclusive society.

## Disclosure statement

The authors declare no conflicts of interest.

## Supporting information


**Appendix** **S1.** Questionnaire for dementia experts

**Table S1.** Types of medical centers for dementia
^†^Conducted in collaboration with other medical facilities

## Data Availability

Research data are not shared. The present study was conducted by Hiroshima University in collaboration with the COVID‐19 response team of the Japan Geriatrics Society, the Japan Society for Dementia Research, and the Japanese Psychogeriatric Society, and no consent for secondary use of the data was obtained from the participants.
