# Supplementary material for: Experts' perception of support for people with dementia and their families during the COVID‐19 pandemic
Source: Geriatr Gerontol Int. 2021 Nov 9;22(1):26–31. doi: 10.1111/ggi.14307 (PMC8653314; doi:10.1111/ggi.14307)
Supplement: Supplementary file 1 — Appendix S1. Questionnaire for dementia experts [file GGI-22-26-s002.docx]

Doc SuppInfo1. Questionnaire for dementia experts

Q１．Please check the appropriate item for the type of the medical center for dementia you belong to.

❑ Core type　　　　❑ Regional type　　　　❑ Collaborative type

Q２．Please check the appropriate item for the type of medical facility you belong to.

　　　　❑ General Hospital　　❑ Psychiatric Hospital　　❑ Clinic

　　　　❑ Other (　　　　　　　　　　　)

Q３．Please check the appropriate item for your clinical department.

　　　　❑ Psychiatry 　❑ Geriatric Medicine　　❑ Neurology

❑ General internal medicine　　　　　❑ Neurosurgery

❑ Other (　　　　　　　　　　　)

**Please share your opinion on the support needed for people with dementia and their families in terms of future COVID-19 measures.**

Q４．It has been pointed out the infection prevention measures—including physical distancing and refraining from going out—may result in worsening of dementia symptoms and decline of cognitive and physical function. What do you think should be done to prevent unfavorable effects of restriction measures (e.g., going out and exercising)? Please tell us your broader opinion, including measures that should be taken not only by the medical center for dementia, but also by the local government and other related organizations.

Q 5．In cases of shutting down or reducing long-term care insurance services due to a sharp increase in the number of COVID-19 infected patients, families took care of people with dementia temporarily instead of these services or felt an increased care burden due to worsening of their symptoms and decline in their physical function.

What measures should be taken to reduce the care burden on families? Please share your opinion, including measures taken not only by the medical center for dementia, but also by the local government and other related organizations.

Q 6. It has been pointed out that people with dementia may have difficulty accessing information and services related to dementia. Do you think that medical facilities such as the medical center for dementia should provide information on COVID-19 and infection control measures for people with dementia? If so, what kind of information should be provided? (e.g., printed and distributed notices from the local government).

Q 7. Households in which only the people with dementia live (e.g., households with a single person with dementia or households in which both husband and wife have dementia) may find it difficult to transition to a new lifestyle to prevent infection and to take measures to prevent infection.

How should we support these households in the future? Please share your broader opinion, including measures taken not only by the medical center for dementia, but also by the local government and other related organizations.

Q 8. It is presumed that COVID-19 infection control measures will require long-term efforts. How should we carry out outreach activities such as initial-phase intensive support teams for dementia and home-visit medical care in the future? Please share your opinions.

Q 9. To maintain the quality of life of people with dementia, it is generally important to interact not only with family members, but also with the local community and peers. However, it is difficult to balance the implementation of infection control measures against COVID-19 with such interactions.

In the future, how should we support people with dementia to maintain their social interactions. Please share your opinions.

Q 10. How would you advise medical or long-term care facilities to respond to an infected person with dementia? Please share your opinions.
