# Supplementary material for: Experts' perception of support for people with dementia and their families during the COVID‐19 pandemic
Source: Geriatr Gerontol Int. 2021 Nov 9;22(1):26–31. doi: 10.1111/ggi.14307 (PMC8653314; doi:10.1111/ggi.14307)
Supplement: Supplementary file 2 — Table S1. Types of medical centers for dementia †Conducted in collaboration with other medical facilities [file GGI-22-26-s001.docx]

Table SuppInfo1. Types of the Medical Center for Dementia

|  | | **Core type** | **Regional type** | **Collaborative type** |  |
| --- | --- | --- | --- | --- | --- |
| Type of medical facility | | Hospital | Hospital | Hospital / Clinic |  |
| Sphere of activity | | Prefectural areas | Secondary medical care areas smaller than prefectural areas | |  |
| Specialty of medical function | Differential diagnosis and consultation | Differential diagnosis of dementia and provision of specialized medical consultation | | | |
|  | Assigning staff (number of staff) | - Dementia specialist or physician with at least five years’ experience in specialized medical care (one or more) - Clinical psychologist (1) - Mental health worker or public health nurse (two or more) | - Dementia specialist or physician with at least five years’ experience in specialized medical care (one or more) - Clinical psychologist (1) - Mental health worker or public health nurse (two or more) | - Dementia specialist or physician with at least five years’ experience in specialized medical care (one or more) - Clinical psychologists, mental health worker, nurse, or public health nurse. (one or more) |  |
|  | System of inspection | - CT - MRI - SPECT^†^ | - CT - MRI^§^ - SPECT^†^ | - CT^§^ - MRI^§^ - SPECT^†^ |  |
|  | Medical treatment for BPSD and physical complications | Secure a dedicated bed for treatment | Cooperation with medical institutions that can provide the medical treatment for acute stage | |  |
|  | Set up a room for medical consultation | Required | | - |  |

†Conducted in collaboration with other medical facilities
